# JAKE® Multimodal Data Capture System: Insights from an Observational Study of Autism Spectrum Disorder

**DOI:** 10.3389/fnins.2017.00517

**Published:** 2017-09-26

**Authors:** Seth L. Ness, Nikolay V. Manyakov, Abigail Bangerter, David Lewin, Shyla Jagannatha, Matthew Boice, Andrew Skalkin, Geraldine Dawson, Yvette M. Janvier, Matthew S. Goodwin, Robert Hendren, Bennett Leventhal, Frederick Shic, Walter Cioccia, Gahan Pandina

**Affiliations:** ^1^Neuroscience Therapeutic Area, Janssen Research and Development, Titusville, NJ, United States; ^2^Computational Biology, Discovery Sciences, Janssen Research and Development, Beerse, Belgium; ^3^Clinical Biostatistics, Janssen Research and Development, Titusville, NJ, United States; ^4^Statistical Decision Sciences, Janssen Research and Development, Titusville, NJ, United States; ^5^Informatics, Janssen Research and Development, Spring House, PA, United States; ^6^Departments of Psychiatry and Behavioral Sciences, Duke Center for Autism and Brain Development, Duke University School of Medicine, Durham, NC, United States; ^7^Department of Psychiatry, Children's Specialized Hospital, Toms River, NJ, United States; ^8^Department of Health Sciences, Northeastern University, Boston, MA, United States; ^9^Department of Psychiatry, School of Medicine, University of California, San Francisco, San Francisco, CA, United States; ^10^Department of Pediatrics, Center for Child Health, Behavior and Development, Seattle Children's Research Institute, University of Washington, Seattle, WA, United States; ^11^Global Digital Health, Janssen Research and Development, Raritan, NJ, United States

**Keywords:** autism spectrum disorder (ASD), biosensor, biomarker, software, assessment

## Abstract

**Objective:** To test usability and optimize the Janssen Autism Knowledge Engine (JAKE®) system's components, biosensors, and procedures used for objective measurement of core and associated symptoms of autism spectrum disorder (ASD) in clinical trials.

**Methods:** A prospective, observational study of 29 children and adolescents with ASD using the JAKE system was conducted at three sites in the United States. This study was designed to establish the feasibility of the JAKE system and to learn practical aspects of its implementation. In addition to information collected by web and mobile components, wearable biosensor data were collected both continuously in natural settings and periodically during a battery of experimental tasks administered in laboratory settings. This study is registered at clinicaltrials.gov, NCT02299700.

**Results:** Feedback collected throughout the study allowed future refinements to be planned for all components of the system. The Autism Behavior Inventory (ABI), a parent-reported measure of ASD core and associated symptoms, performed well. Among biosensors studied, the eye-tracker, sleep monitor, and electrocardiogram were shown to capture high quality data, whereas wireless electroencephalography was difficult to use due to its form factor. On an exit survey, the majority of parents rated their overall reaction to JAKE as positive/very positive. No significant device-related events were reported in the study.

**Conclusion:** The results of this study, with the described changes, demonstrate that the JAKE system is a viable, useful, and safe platform for use in clinical trials of ASD, justifying larger validation and deployment studies of the optimized system.

## Introduction

Autism spectrum disorder (ASD) is a heterogeneous group of neurodevelopmental disorders characterized by deficits in social communication and restricted and repetitive behaviors (American Psychiatric Association, 2013). The prevalence of ASD in children is estimated to be ~1% worldwide (Elsabbagh et al., 2012a; Centers for Disease Control Prevention (CDC)., 2016). In the United States, the overall estimated ASD prevalence is 14.6 per 1,000 children according to the most recent surveillance data from the Center for Disease Control's Autism and Developmental Disabilities Monitoring Network (Christensen et al., 2016).

Therapies for the symptoms of ASD are currently limited to behavioral interventions and medications that target comorbid symptoms (e.g., obsessive-compulsive behavior, hyperactivity, irritability, anxiety, depression). There is no approved agent that effectively treats the core symptoms of ASD or improves the natural history of the condition. Furthermore, development of novel treatments that target core symptoms of ASD, beginning with study in proof-of-concept clinical trials, is limited by substantial biological and clinical heterogeneity, lack of a unified sensitive and objective endpoint for core symptoms, and difficult-to-quantify risk of potential false negative results, among other factors (Ghosh et al., 2013). A system that addresses these operational complexities could utilize various experimental markers identified by the research field, and integrate them with broader phenotyping tools.

The purpose of this report is to describe the Janssen Autism Knowledge Engine (JAKE®), which is being developed to provide quantifiable and reproducible measures for use in treatment monitoring and identification of ASD subgroups. Suitable measures for JAKE were identified through review of existing research using biosensors in ASD (Pelphrey et al., 2002; Wang et al., 2004; Grice et al., 2005; Murias et al., 2007; Coben et al., 2008; de Wit et al., 2008; Sasson et al., 2008, 2011; Moscovitch et al., 2011; Pitcher et al., 2011; Chawarska et al., 2012; Elison et al., 2012; Elsabbagh et al., 2012b; Moore et al., 2012; Tierney et al., 2012; Tye et al., 2013; Wagner et al., 2013) and through consultation with experts in each field. We report herein findings from the first observational, proof-of-principle study of the complete JAKE system conducted in individuals with ASD, and focus on the usability and feasibility of the system. Another aspect of this study, the validity and reliability of the Autism Behavior Inventory (ABI) as compared with existing measures is reported elsewhere (Bangerter et al., 2017). ABI is a parent-reported measure of core and associated ASD symptoms, developed specifically to measure change in behavior during the course of an intervention.

This line of research by our group and parallel work by others [e.g., EU-AIMS (European Autism Interventions^1^); Autism Biomarkers Consortium for Clinical Trials–ABC-CT (Foundation for the National Institutes of Health^2^)] seeks to identify biomarkers that stratify the ASD population according to distinct biological subtypes, thereby providing the foundation upon which efficacy signals in specific responder subgroups can be detected. In contradistinction to the EU-AIMS and ABC-CT initiatives, which are exploring various tools administered by highly trained clinical research professionals in a laboratory setting, the aim of JAKE is to employ a mobile and web-based application coupled with biosensors that can be used by a community-based clinician and even by non-medical persons who can be trained to use them in a general clinical or clinical research setting. This has some similarities to an approach described by Billeci, also in the early stages of development (Billeci et al., 2016). The different components of JAKE, as described further below, are designed to capture sufficiently variable phenotypes and behaviors across all key domains of ASD, while providing a high level of utility—effectively making it simple for caregivers and other observers to record critical information on improvement or worsening of symptoms and behaviors.

### What is JAKE?

**Figure 1:**
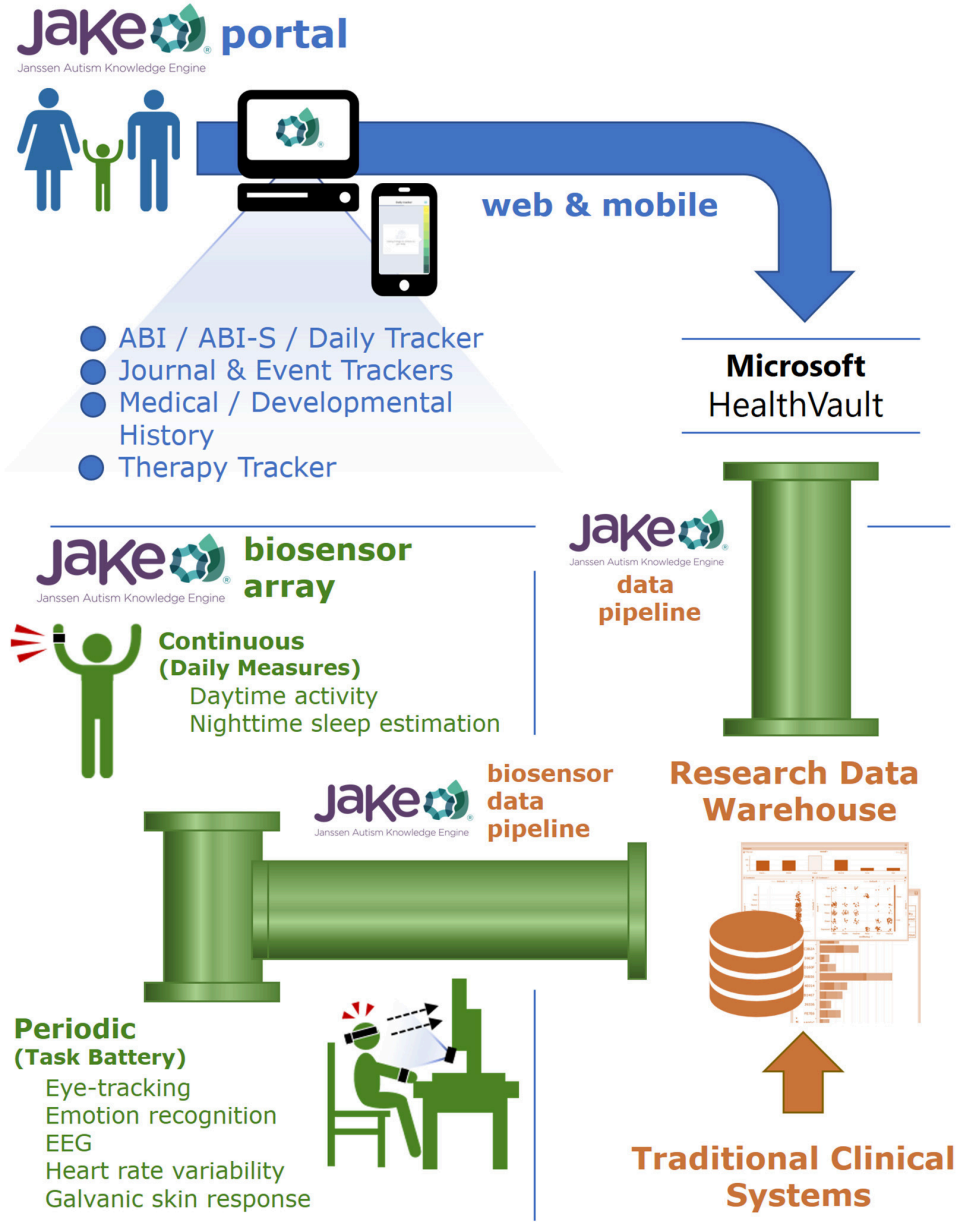
Janssen Autism Knowledge Engine.

JAKE, a dynamically updated clinical research system, consists of several major components (Figure 1), each described below. Inputs from the JAKE Portal and the JAKE Biosensor Array all feed through the JAKE Data Pipeline, where raw data are archived and feature extraction occurs. Finally, cleaned data and analyses are stored in the Janssen Research Data Warehouse (Janssen RDW) and combined with traditional clinical trial databases.

### JAKE portal

The JAKE Portal and mobile apps, both native Android and iPhone iOS apps, utilizes Microsoft HealthVault (“HealthVault”) as its primary data backend, reading and writing health information only to this record. HealthVault, a publicly available electronic personal health record system, is used to store consumer health data, interact with health applications such as electronic medical records (EMRs), and authorize sharing of information across caregivers and health care providers. The use of HealthVault permits the participant or caregiver to retain access to any data entered using the JAKE Portal, even after the study has ended. The system is modular and includes robust application protocol interfaces (APIs) allowing for the potential to write data to other back-end data store systems.

HealthVault servers are located in controlled Microsoft facilities, in physically secured cabinets (HealthVault FAQs, 2015). The HealthVault platform has been the subject of security testing by both Microsoft and third parties, including “white hat hacker” penetration testing. Connections between the user and HealthVault (and the JAKE Portal) are encrypted using strong industry-standard methods.

Described below are several of the key components of the JAKE Portal.

The JAKE Portal, accessible through most web browsers and mobile devices, encompasses tools and technologies designed for use by clinicians and caregivers to log symptoms, record interventions, and track progress. Each of the JAKE Portal modules is described below.

The *Dashboard or JAKE Portal home page* provides a detailed overview of the participant's record: displaying upcoming appointments and study-related tasks and notifications.

The *Medical/Developmental History module*, filled out by caregivers, collects a participant's comprehensive developmental and medical history. Key sections include a complete family and demographic history, gross/fine motor, language, and social development milestones, current providers and therapies, medications and supplements, as well as many others. In addition to this electronic record, all information gathered by the module is printable, allowing the participant or caregiver to share paper copies of their medical histories easily with new health care providers.

**Figure 2:**
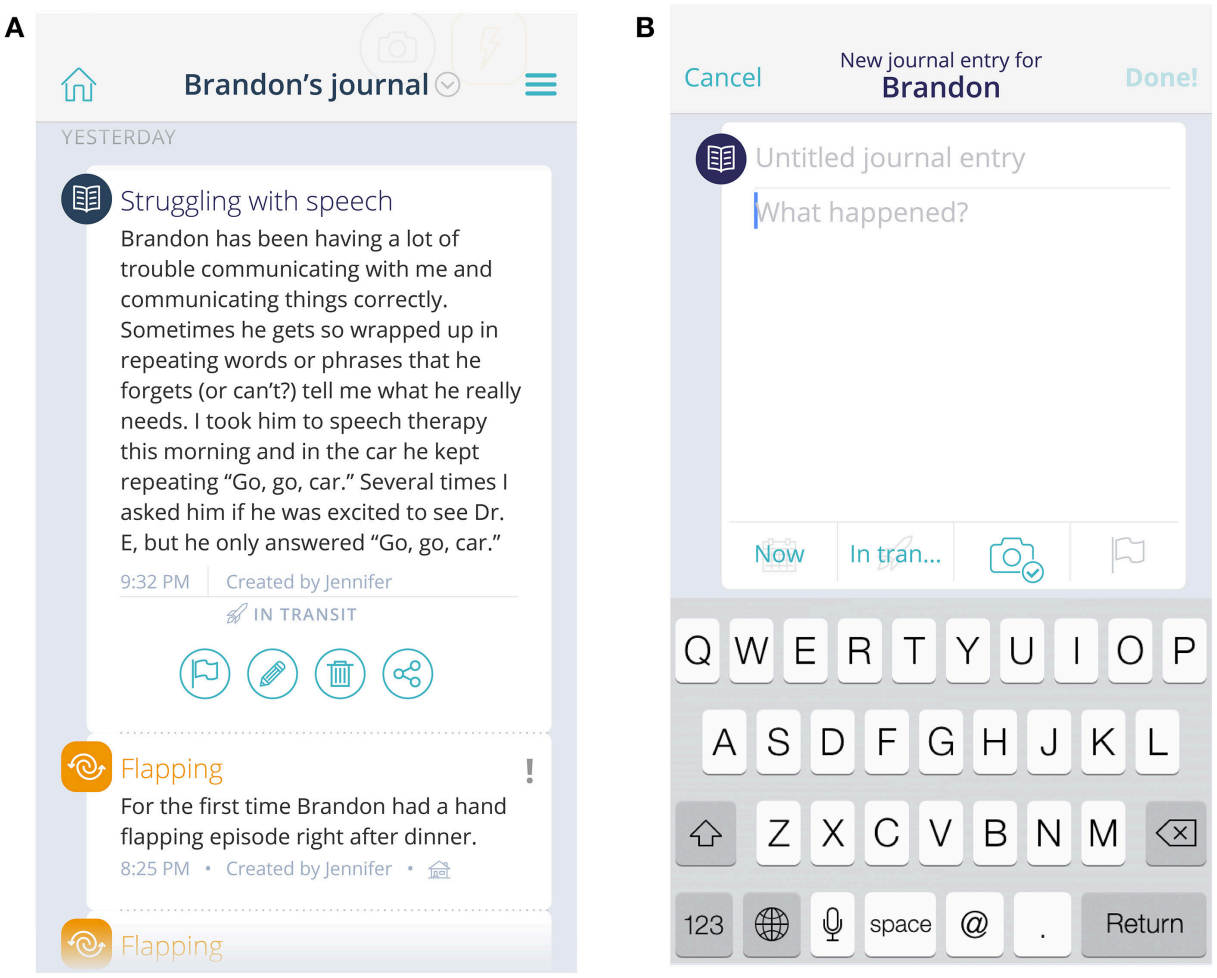
View of journal and event entries **(A)** and new journal entry field **(B)**. The child's name and journal/event entries are fictional, not original.

A *Journal*, containing a semi-structured, free-text field, is available and can be used to document daily behaviors. The journal can help by providing contextual information to situations—such as highlighting a positive event or identifying a particular location where a problem behavior always seems to surface. Entries are stored in chronological order. Journal entries also become visible as a hover field on the Dashboard chart, allowing visualization against behavior tracking data. Finally, the Journal can serve as a method of communication across caregivers and care providers—users can filter entries to include just their own or view all entries created about the child on whom they are reporting (Figures 2A,B).

**Figure 3:**
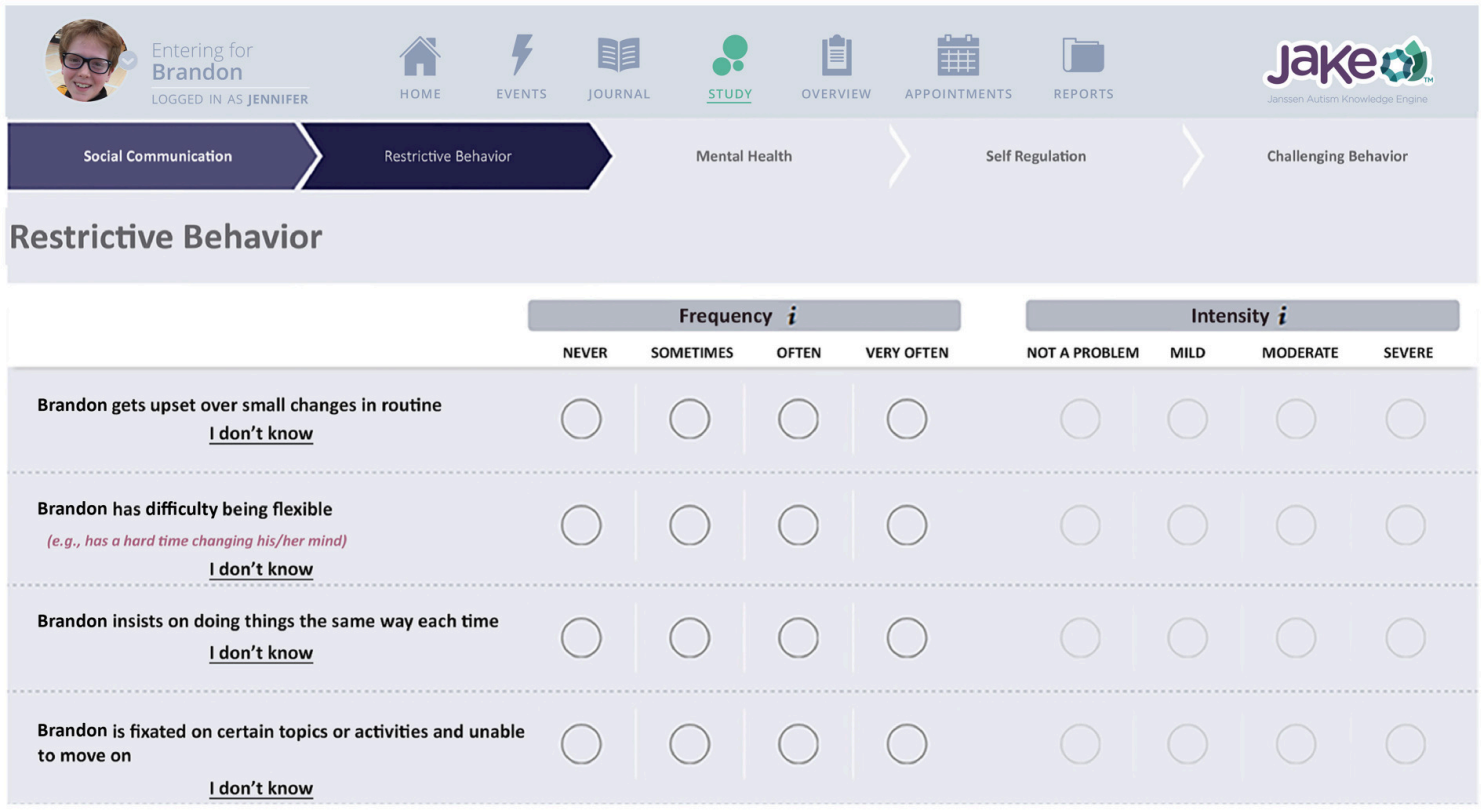
Chart indicating biosensor features. Written, informed consent was obtained from the parents for publication of their child's image. The child's name is fictional.

The *ABI* is an online rating scale that assesses core and associated symptoms of ASD, Figure 3, Bangerter et al., 2017). The ABI can be downloaded in the USA from https://www.janssenmd.com/ (in the tools/psychiatry section) and accessed outside the USA via email request to autismbehaviorinventory@its.jnj.com. In conjunction with related components of JAKE, it is designed to capture variable clinical presentations across all key ASD core and associated domains, while providing a high level of utility—effectively making it simple for caregivers and other observers to record critical information on improvement or worsening of symptoms and behaviors. Scale development began with a group of 160 questions, which were tested and subjected to factor and usability analyses, resulting in a final set of 97 questions. This set of questions was further tested and fine-tuned in this study (results reported by Bangerter et al., 2017). The ABI is intended to be completed at weekly intervals, or longer. Additionally, parents were presented with a subset of the behaviors to rate every day (*Daily Tracker*).

**Figure 4:**
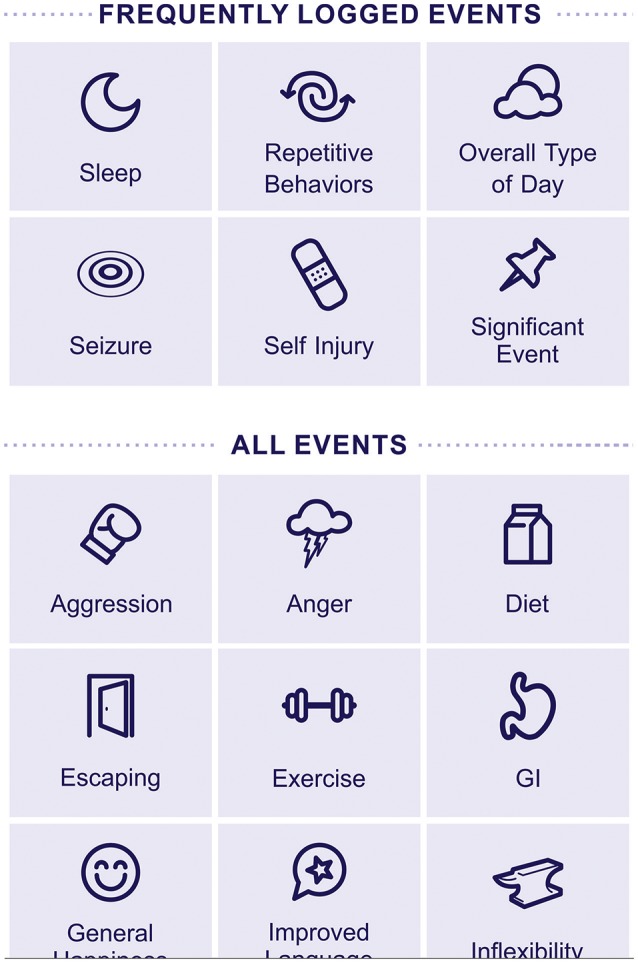
Event tracker screen—mobile version.

Companions to the ABI are an *Event Tracker* (example screen shot shown in Figure 4) and a *Therapy Tracker*. In these modules, caregivers track events (tantrum, stereotypy, social interaction, etc.) and medical and other therapies, as well as medications, diet, and illnesses.

### JAKE biosensor array

The JAKE Biosensor Array includes selected biosensors to assess physiological characteristics and behavior related to the core symptoms of ASD. The JAKE Biosensor Array is divided into two primary components: a set of continuous, wearable biosensors that gather information on a daily basis, and a set of periodic biosensors designed to gather feedback during a battery of experimental tasks administered via computer in a lab setting.

Continuous biosensors and key parameters collected by each include: (1) wrist-based accelerometry/actigraphy with the aim of measuring the frequency and durations of repetitive motor movements or patterns and activity during the day; (2) similar actigraphy measures, in conjunction with ambient light sensors, with the aim of assessing sleep onset, quality, and duration; and (3) electrodermal activity (EDA) sensors, designed to measure autonomic nervous system arousal.

Periodic biosensors are used only during the time that a participant is exposed to specific visual stimuli via a computer screen interface. The key biological measurements collected include electroencephalography (EEG), eye-tracking, and electrocardiography (ECG). Table 1 includes examples of the types of features that were extracted from the periodic biosensors. The experimental tasks and stimuli (the JAKE Task Battery) used with the periodic biosensors include tasks designed specifically to observe differences between ASD and typically developing (TD) children. While all periodic biosensors are used throughout administration of the Task Battery, several tasks focus on a particular measurement, as described below. EDA is also collected throughout the periodic task battery.

**Table 1:** Example features extracted from periodic biosensors.

**Eye-tracking**	**EEG**	**ECG**	**EDA**
Tobii X2-30, B-Alert® X24	B-Alert® X24	CamnTech Actiwave Cardio Single-Channel ECG	Q™ Sensor
General features: Fixations and saccades (velocity based binocular algorithms) % valid time on screen % on ROIs (also normalized to % valid time) Pupil size Specific features: Biological Motion preference (%), first saccade orienting (%]) saccade latency, fixation orienting (%), fixation latency VET exploration, preservation, detail orientation, RQA features	Induced EEG activity (estimated for each electrode and different brain region) Power spectra at different bands (delta, theta, alpha, beta, gamma) Normalized power spectra at different bands Brain asymmetry index for different bands Coherence between at different bands ERP: Components' amplitudes with peak- and area-based methods Components' latencies with peak- and area-based methods	HR SDNN SDSD rMSSD NN50 pNN50 ApEn LF HRV Normalized LF HRV HF HRV Normalized HF HRV LF/HF HRV	Tonic activity: SCL Phasic activity: SCRR

*ApEn, approximate entropy; ECG, electrocardiography; EEG, electroencephalography; EDA, electrodermal activity; ERP, event-related potentials; HF, high frequency; HR, heart rate; HRV, heart rate variability; LF, low frequency; NN50, number of pairs of successive NNs that differ by more than 50 ms; rMSSD, root mean square of successive differences; RQA, recurrence quantification analysis; pNN50, proportion of NN50 divided by total number of NNs; RQA, recurrence quantification analysis; SCL, skin conductance level; SCRR, skin conductance response rate; SDNN, standard deviation of normal to normal R-R intervals; SDSD, standard deviation of successive differences; VET, visual exploration task*.

EEG is primarily examined during presentation of: (1) a video designed to elicit a resting state (Wang et al., 2013), in which a participant watches sand falling through an hourglass for 1 min and 30 s and then rests with eyes closed for the same duration; (2) short videos [comprised of clips of dynamic social stimuli (faces and bodies) and non-social stimuli (objects and scenes)] (Pitcher et al., 2011); (3) a set of photographs depicting different facial expressions (Pelphrey et al., 2002; Wang et al., 2004; Tottenham et al., 2009; Moore et al., 2012; Sepeta et al., 2012; Wagner et al., 2013); and (4) full color photographic images of different human faces directing their gaze straight at the viewer (direct gaze) or averted to either right or left (averted gaze) (Grice et al., 2005; Elsabbagh et al., 2012b; Tye et al., 2013).

Eye-tracking is primarily examined during: (1) video of a woman engaging in child-directed speech, an upward age extension of stimuli by Chawarska et al. (2012) that includes bids for joint attention (Campbell et al., 2014; Plesa-Skwerer et al., 2016); (2) the Visual Exploration Task (VET) (Sasson et al., 2008, 2011; Elison et al., 2012) which contains 12 arrays of 24 images each [including two sets each of social images, high autism interest (HAI), and low autism interest (LAI) objects], presented for 10 s, where the participant is free to explore the array while the eye-tracker monitors the participant's visual attention; (3) a Biological Motion Preference task [based on the work of Annaz et al. (2012) and Jones et al. (2008); see Shic et al. (2014a, 2015)], which contains two side-by-side videos of moving dots, one moving in human fashion (biological motion) and the other moving randomly (non-biological motion) in random left-right order; and (4) an Activity Monitoring task (Shic et al., 2011, 2014b) in which a participant views a video recording of human actors performing a social activity, with visually salient distractors in the background.

ECG is monitored during the entire battery and assesses characteristics such as heart rate (HR) and features derived from it [e.g., heart rate variability (HRV)], with attention to abnormalities consistent with impairments in the autonomic nervous system.

To measure cognitive function, the Cogstate Computerized Test Battery (2016) was included in the JAKE Task Battery and evaluated in this study. Involved are tests of information processing speed and fine motor skills, visual attention, visual recognition memory, working memory, and emotion processing, to assess ASD-associated neurocognitive deficits (Baron-Cohen et al., 2001; Surowiecki et al., 2002; Bavin et al., 2005; Mollica et al., 2005; Crutcher et al., 2016).

The JAKE Task Battery also assesses participant's facial affective expression and physiological reactivity to sensory, social, and emotional stimuli. For the former, a standard web camera and the iMotions® Emotient™ FACET (2016) software module are used to analyze facial expressions across all task presentations (participant affective expression), with analyses particularly focused on facial expressions elicited by the presentation of a set of videos chosen for their humorous visual content. In addition, the participant is prompted to produce specific emotional facial expressions in response to words such as “happy” and “sad,” which are analyzed by FACET.

The JAKE Biosensor Workbench (JBW) is a management software / hardware platform used for administration of the Task Battery. The iMotions® Biometric Research Platform software (formerly Attention Tool) is used to synchronize data streams from several periodic biosensors, while presenting the previously described stimuli. Additional synchronization for the EEG is provided using the Cedrus StimTracker™. Exported results are merged with offline data sources, curated, and then transmitted directly to the JAKE Data Pipeline via a secure file transfer protocol application. The result is time-synchronized integration and comprehensive analysis of all biosensor data inputs, in combination with manually-entered data extracted from the JAKE Portal.

### JAKE data pipeline

The main objectives the JAKE Data Pipeline addresses are data extraction, filtering by identified fields of interest, de-identification of certain free-text fields such as journal entries, data cleanup, and harmonization, archiving of all raw data in order to establish an audit trail, fixing identified data inconsistencies, transforming the native HealthVault XML-formatted data into relational comma-separated values (CSV) tables better suitable for data analysis, and finally delivering output data stream.

### Janssen research data warehouse (RDW)

The Janssen RDW is a set of tools, data stores, and data feeds designed for robust retrieval, storage, and analysis of all final, cleaned data generated within the JAKE system. Data residing in the RDW can be mined using fit-for-purpose analytic tools and strategies to track treatment outcomes and assess symptom patterns and subpopulations. The data are also being used to develop new analytic software to interpret significant biosensor data events. Following a manual de-identification procedure, subsets of information contained in the Janssen RDW may be shared with researchers and members of the scientific community. RDW's data flow consists of feeds, pre-processing, feature extractions, transformations, and outputs.

Data feeds include: (1) the JAKE Data Pipeline for the HealthVault-originated data; (2) a secure file transfer protocol server for collecting biosensor data; (3) an electronic case report form (eCRF) assembling information on a participant's visits and basic demographic data; (4) internal clinical data repositories; (5) experts' assessments on data quality created using specially-designed data visualization tools; and (6) transferring paper-based rating scale data collected in traditional eCRFs. Most of the feeds are automated and executed overnight.

### Early investigation of JAKE

A prospective observational study was conducted at three sites (two of which are academic universities and the other, a clinical service provider) to test and optimize the platform components of the JAKE system, as well as the selected biosensors and procedures used for objective measurement of core and associated symptoms of ASD.

Independent Review Boards (Duke University Health Systems Institutional Review Board, Durham NC and Western Institutional Review Board, Puyallup, WA) approved the study protocol and its amendments. The study was conducted in accordance with the ethical principles originating from the Declaration of Helsinki, consistent with Good Clinical Practices and applicable regulatory requirements. Parents (or legal guardians) of all participants provided written informed consent and minors provided written informed assent before enrolling in the study. The study is registered at clinicaltrials.gov, NCT02299700.

## Materials and methods

### Study population

The study enrolled children and adolescents with a diagnosis of ASD according to Diagnostic and Statistical Manual of Mental Disorders 5th edition, DSM-5 (American Psychiatric Association, 2013), at least a minimum of mild rating on the Child Autism Rating Scale 2 [CARS-2 (Schopler et al., 2010)], and a measured composite score on the Vineland Adaptive Behavior Scale II [VABS-II (Perry and Factor, 1989)] ≥60, the latter [as a proxy for intelligence quotient (IQ)] to maximize the likelihood of completion of the task battery. ASD participants were permitted to receive behavioral and/or pharmacologic intervention for ASD and comorbid disorders during the course of the study.

TD children with similar demographics (based on sex, age, and race) compared to ASD participants were also enrolled. The TD children had a score in the normal range on the Social Communication Questionnaire (SCQ), had no existing DSM-5 major mental health disorder according to the Kiddie-SADS-Present and Lifetime Version (K-SADS-PL) (Kaufman et al., 1997), and were not taking psychotropic medications. The TD cohort provided normative data for comparison with that from ASD participants.

Autism Diagnostic Observation Schedule (ADOS) and Autism Diagnostic Interview-Revised (ADI-R) were not used for diagnosis and intelligence quotient (IQ) was not captured in this pilot study, but will be included in future validation studies. There are also validation plans across a broader range of functioning levels, which should be possible due to the passive viewing nature of most of the tasks.

### Study design

The usability of and methods to increase compliance with biosensors were developed and tested at two sites (Duke University Medical Center, Durham, NC; Northeastern University, Boston, MA) over a 1-week period and at one clinical site (Children's Specialized Hospital, Toms River, NJ) at a single visit for a subset of participants. Functionality of JAKE was evaluated at all three sites (over a 4-week period at Children's Specialized Hospital and over 1 week at the other two sites).

Wearable biosensors tested in this study included: (1) child daytime sensor (Q™ Sensor)—a wireless wristband biosensor worn during all waking hours that recorded changes in EDA, skin surface temperature, and 3-dimensional acceleration; and (2) child nighttime sensor (AMI Micro Motionlogger Sleep Watch) worn during night-time sleep.

#### Periodic sensors

EEG data were collected using B-Alert® X24, a wireless, wet electrode system that allows participants to move freely. ECG data were collected using the CamNTech Actiwave Cardio Single-Channel ECG. Eye-tracking data were collected using Tobii X2-30.

Measures and tasks were specifically chosen to be appropriate across ages (6 years old to adult) and a broad range of cognitive functioning (>60 on the VABS). Most tasks were passive viewing for this purpose. Clinical judgment was used to determine whether participants could complete the eyes-closed part of the task, and the Cogstate battery appeared at the end of the work bench, allowing for opting out as necessary. Additional breaks were possible for those who needed them. The cutoff on VABS was implemented to ensure a level of functioning high enough to be capable of completing the workbench.

Given the length of the battery (included a broad range of tasks to enable reduction/refinement, based on performance, in future versions of JAKE), tasks split into three sets.

Parents completed an exit survey, which assessed the usability of JAKE. Questions were answered on a 5-point Likert scale (e.g., 1 = very difficult, 5 = very easy).

### Study results and discussion

The study population comprised 29 children and adolescents with ASD and 6 TD children. A large ASD cohort was deliberately enrolled to determine the feasibility of administering JAKE to a heterogeneous group of individuals with ASD; a much smaller number of TD participants were enrolled to gather comparative data.

The majority was male and white; their mean age was 10 years (Table 2). At screening of the ASD participants, the mean [standard deviation (SD); range] CARS-2 total score was 47.2 (8.45; 24–62) and VABS total score was 73.3 (17.77; 24–111).

**Table 2:** Demographic characteristics of study participants.

	**Autism spectrum disorder *N* = 29**	**Typically developing children *N* = 6**
**SEX, N (%)**		
Male	25 (86.2)	4 (66.7)
Female	4 (13.8)	2 (33.3)
**AGE**		
Mean (SD)	10.1 (5.20)	10.0 (2.83)
Range	4–27	7–14
**RACE, N (%)**		
White	27 (93.1)	6 (100)
Black	0	0
Multiple	2 (6.9)	0

Twenty (14 ASD and all 6 TD) of these participated in the JBW.

### Experience with the JAKE system

Our study was designed to establish the feasibility and utility of the JAKE system and to learn practical aspects of its implementation; therefore, we do not present results of group differences between TD and ASD or differences correlating severity of ASD based on sensor data.

The JAKE system was successful in gathering responses to the ABI (Bangerter et al., 2017). It also proved feasible for collecting medical-developmental history of participants, journal entries, and, to a limited extent, ASD events (such as parent-identified instances of restricted and repetitive behaviors).

Although data acquisition from the ECG device was excellent, issues with our EEG and eye-tracking led to only limited data availability. In no cases were we successful in capturing all sensor data types in a single participant or across both time points. Changes to mitigate these deficiencies are described in the following section.

We present here key aspects of our analytical process and examples of results from individual participants to showcase expected findings using the complete system.

**Figure 5:**
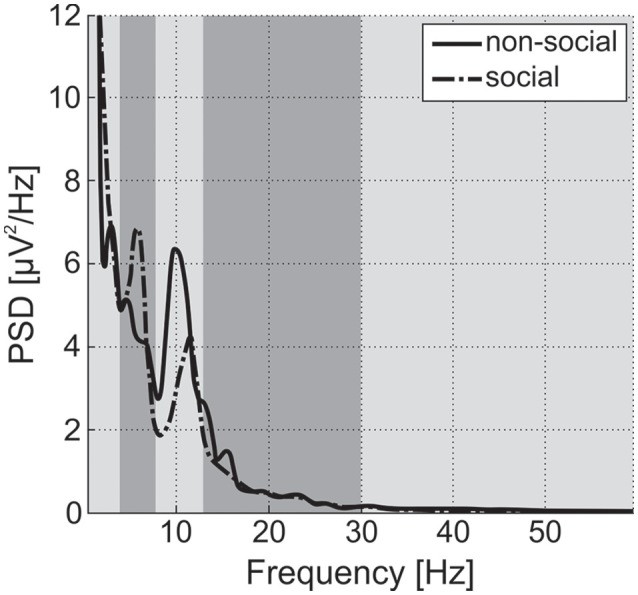
Power Spectral Density (PSD) for social and non-social stimuli averaged among central electrode positions for a single TD participant from the study. Vertical strips denote delta, theta, alpha, beta, and gamma bands. Alpha power is suppressed and theta power is enhanced during observation of social stimuli in comparison to non-social ones. For ASD participants, differences in alpha and theta are expected to be inversed or negligible (Dawson et al., 2012).

**Figure 6:**
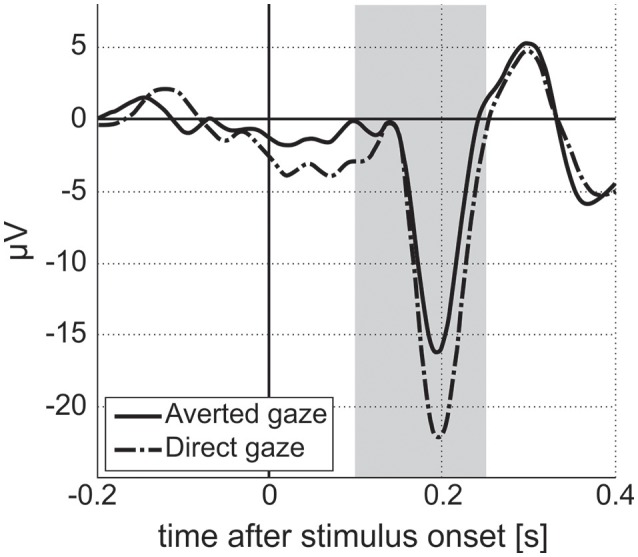
Event-related responses averaged along parietal electrodes for facial stimuli with averted and direct gazes for a single ASD participant from the study. Vertical stripe indicates interval for N170 estimation. Amplitude of N170 component for averted gaze stimuli is lower than that for direct gaze stimuli. We expect to see differences in amplitude/latency of N170 between two stimuli conditions in the ASD population, with no differences for TD participants (Grice et al., 2005).

EEG recordings were checked to validate that Power Spectrum Density (PSD) followed a 1/f shape (“pink noise”), with possible experiment-related band-specific induced activity [example shown in Figure 5 (Dawson et al., 2012)]. PSD was estimated following Welch methods in 4 s segments with 75% overlap convolved with a Hamming window. For averaging of PSD data, it was requested to obtain at least 30 such artifact-free segments (with a smaller number for NIMSTIM Emotional and Biological Motion Preference Tasks) during which the participant paid attention to the stimulus/screen as demonstrated by the eye-tracking data (excluding the eyes closed condition). For averaging of ERP data, it was requested to obtain at least 50 (out of 150) baseline-corrected (baseline: from –200 to 0 ms before correspondent stimuli onset) responses to stimuli per condition (zero-phase band-pass filtered between 0.1 and 30 Hz) without artifacts and with attention to stimulus (as demonstrated by eye-tracking data). The possibility of detecting an N170 component in (100 250) ms after stimulus onset was evaluated [example shown in Figure 6 (Grice et al., 2005)].

For eye-tracking, participants were asked to look at the screen but were not provided with specific instructions on where to look. A threshold of 50% was applied, with data being considered not valid, and excluded if the participant was not looking at the screen for more than half of the time a task was running. Calibration mapping of participants' detected eye positions to points on the screen were conducted at the beginning of each 15 min experimental block using a 5-point calibration procedure.

**Figure 7:**
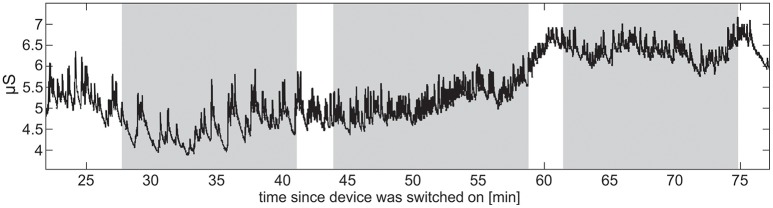
Raw EDA data from a single TD participant. The vertical stripes denote the three sets into which the stimuli were divided. Increase in SCL indicates the participant's increasing arousal toward the end of the task battery.

Raw EDA data were assessed in terms of amplitudes, dropped signals, sensitivity of measuring skin conductance level (SCL, tonic activity), and skin conductance responses (SCR, phasic activity) (exemplar recordings shown in Figure 7). Sensitivity for capturing phasic activity was participant-dependent, potentially related to density of eccrine sweat gland differences on the wrist across participants. It was also noticed that, independent of the experiment, SCL increased for about 30 min after the device was placed on a wrist before the signal stabilized, presumably due to time necessary to establish a consistent moisture barrier.

**Figure 8:**
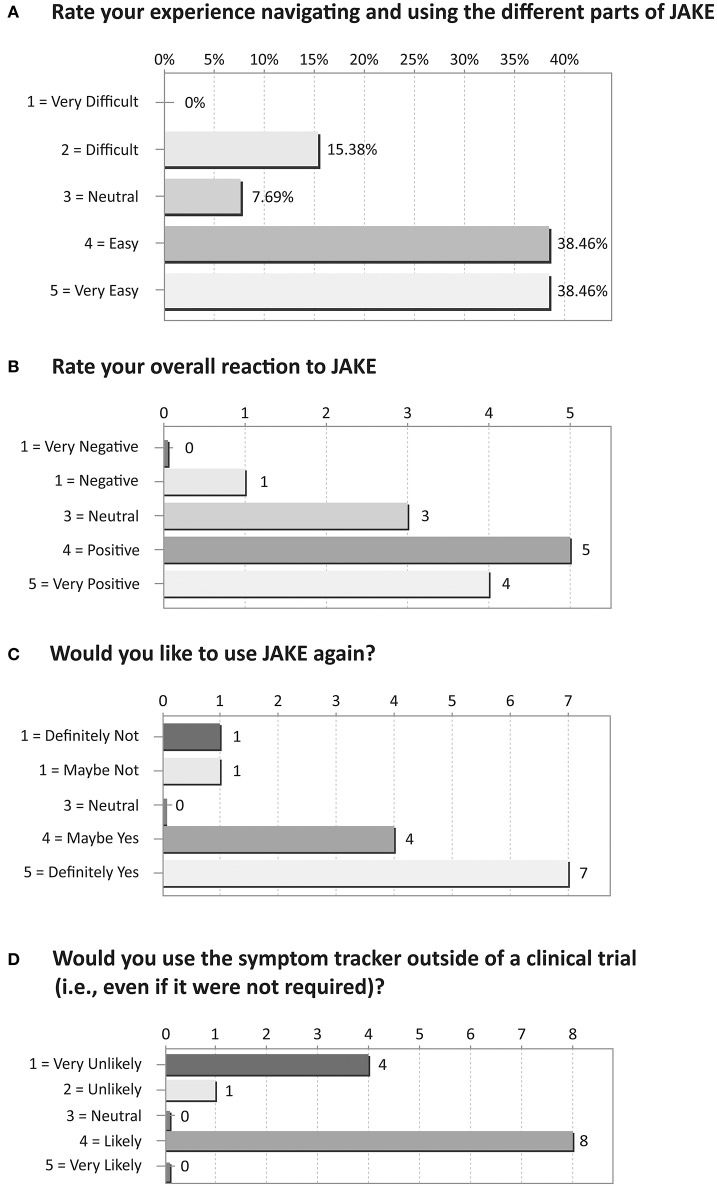
Results of exit survey completed by parents/caregivers of ASD children. **(A)** Rate your experience navigating and using the different parts of JAKE. **(B)** Rate your overall reaction to JAKE. **(C)** Would you like to use JAKE again? **(D)** Would you use the symptom tracker outside of a clinical trial (i.e., even if it were not required)?

Based on their responses to an exit survey, the majority of parents rated their overall reaction to JAKE as positive/very positive, with most finding navigation through the different parts of JAKE easy/very easy (Figure 8).

### Safety

Overall, the incidence of adverse events was low during the study. Adverse events were typical of those in people with ASD, and not related to the JAKE system. Five adverse events were reported for four subjects (i.e., two events of upper respiratory tract infection and one event each of aspiration, pyrexia, and tooth infection). No significant device-related events were reported in the study.

### Resolution of operational challenges and modification of JAKE system

Based on feedback and observation during the study, several activities focused on identification and resolution of operational and quality issues to ensure data integrity, protocol compliance, and safety of study participants. The design learnings based on our experience from the study were used to modify the JAKE system in order to improve reliability in future studies (key examples provided below).

### Jake mobile and portal

Modifications to the JAKE mobile and portal application were made following feedback and results of data collection during the study.

**Figure 9:**
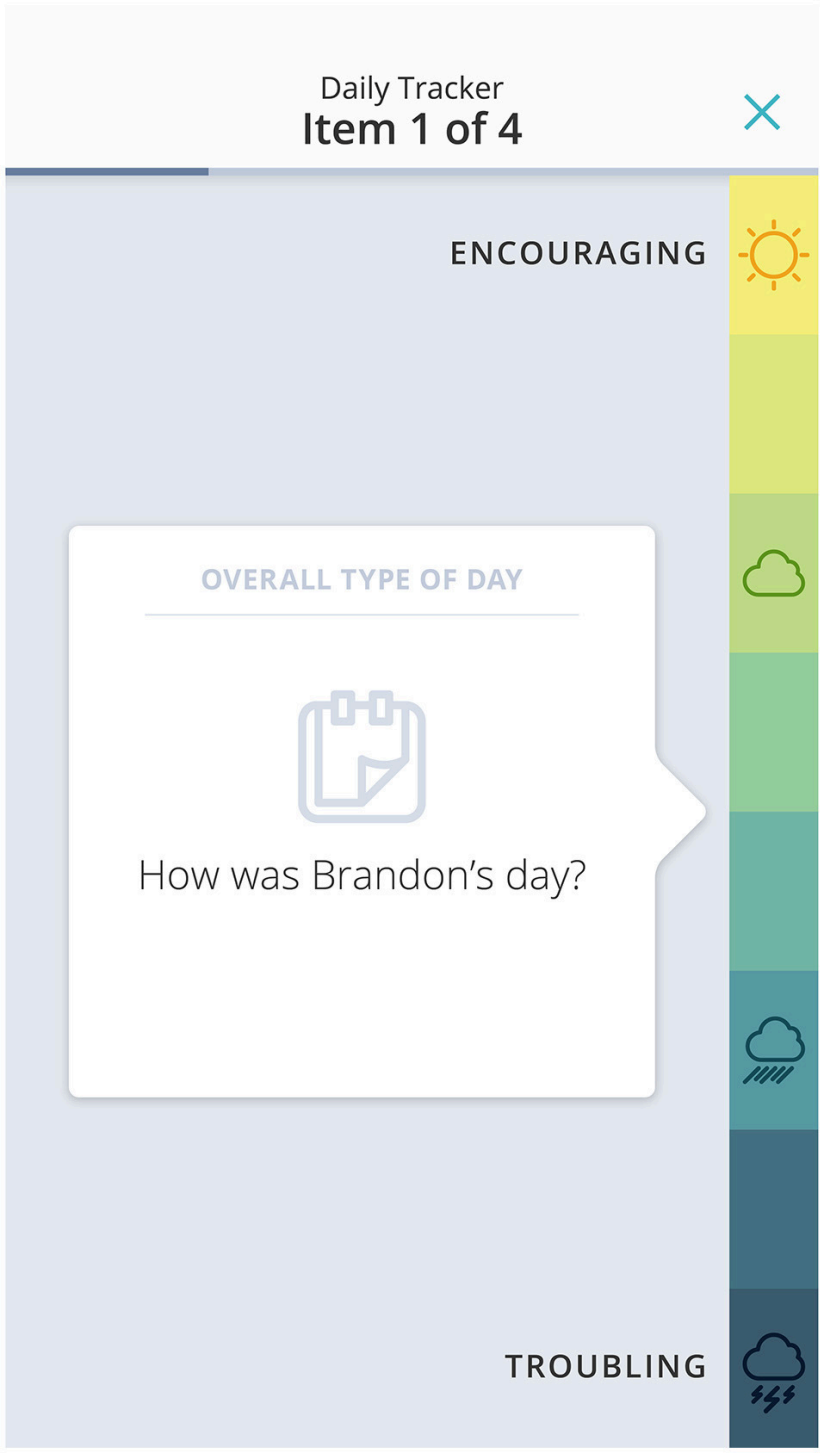
Revised daily tracker. The child's name and tracker field are fictional.

The JAKE Daily Tracker was designed to enable caregivers to report changes in symptoms and behaviors on a daily basis. In this study, the Daily Tracker was made up from a subset of ABI items. Around five items representing a domain, plus an overall question about that domain, were presented each day in a similar format to the questions on the ABI. Following feedback from parents that these daily questions felt repetitive, and were not always relevant to their child, we developed and introduced a new approach that enabled parents to select and track behaviors most relevant for them. The anchors were changed from the ABI anchors to an 8-point sliding scale, which was simple and quick to complete (Figure 9) and had a different feel to the scales parents were completing on a less regular basis. The number of items to complete each day was reduced to three, plus a general overall type of day rating, and this reduced parent burden for completion.

To increase feedback to and engagement of the caregivers, a “journey” chart was added to the JAKE Portal home page, enabling parents to receive feedback on items they entered and look for relationships between reported behaviors and events. It is the primary item on the page and shows a visualization of a participant's overall type of day, sleep, and three selected behaviors from the Daily Tracker over time. All five of these items from the Daily Tracker are plotted on the same chart for the past 10 days, though the end user has the ability to turn “on” and “off” specific lines to focus more on one or two items at a time.

In order to further validate the changes made to the system, two sets of usability tests were conducted on JAKE. Usability testing is typically conducted to obtain impartial user feedback on a system or system design. This feedback, taken as a whole, can be collected to identify minor user interface/user experience (UI/UX) improvements or to determine the direction of future capabilities/features. Both rounds of JAKE usability testing included 10 participants of varying demographics. The tests were all conducted as one-on-one sessions, lasted 1 h and were facilitated by an external partner who specializes in usability testing. The target audience for all sessions was a parent of a child diagnosed with ASD. Scripts were used to ensure consistency across the tests, but the facilitator was also able to probe for more information when the user indicated that something was confusing or unclear. Detailed reports were prepared summarizing the feedback and UI/UX recommendations based on the comments.

Feedback indicated that the charts would provide motivation to the users, and further justified incorporation into the JAKE Portal and apps. Feedback was also obtained on the proposed change of the Daily Tracker to an 8-point scale, and the ability for parents to select their own behaviors to track. This was viewed positively by the participants. The color range and additional icons were also updated as a result of the usability testing.

Additional user testing led to the inclusion of first use cards to help with chart explanations, improved navigation for the app, and changes in the order of items for tracking.

We found that caregivers did not frequently enter ASD events, and when they were entered participants were not always wearing the actigraph/EDA device, thus making it impossible to correlate specific events with sensor data. To mitigate this, we added (for the next study) a 15-min caregiver observation period, twice weekly, wherein the caregiver is instructed to concentrate on entering any ASD events and to ensure participants are wearing the actigraph/EDA sensor.

We also found that there were instances of personally-identifiable information (PII) being transmitted to the sponsor's databases requiring manual deletion. De-identification—the process of removing PII from JAKE Portal extracts—was therefore improved as follows: (1) certain fields in the JAKE Portal were ignored entirely by the JAKE Data Pipeline; and (2) fields where PII could inadvertently be stored were targeted for automatic scrubbing, using the DeID utility (PhysioNet, 2016).

Use of Zweena, a third-party data collection service provider, to help fill out the Medical/Developmental history proved beneficial and was continued.

### Data review system

A set of tools was created for visual exploratory assessments of the JAKE-originated HealthVault data and continuous biosensor data. This included a web-based Site Managers Dashboard to assess adherence and automatic detection of protocol deviations on caregiver data entry tasks and tools to show synchronized data streams on one plot (for example, overlaying raw accelerometer data with the JAKE-reported events). Automatic data quality and integrity checks were introduced into the biosensor data processing pipeline. A dashboard was also created for team members to monitor biosensor data transfers, track data integrity, and provide initial assessments of data integrity. Additional checks were added to ensure the integrity of calculated data features.

### Autism behavior inventory (ABI)

The ABI performed well, demonstrating good reliability and validity (Bangerter et al., 2017). The number of items was reduced slightly to 73 based on correlations from this study and small changes were made in wording or examples for items that parents reported having difficulties understanding.

Clinician investigators had difficulty answering many of the items on the full ABI tracker as they required in-depth knowledge of the participant's daily life. “I don't know” responses to the ABI were rare for parents and more frequently given by clinicians. As a consequence, it was decided that site clinicians would only complete a brief (short) version of the ABI (ABI-Short or ABI-S), which was reviewed to ensure that someone who had only limited contact with the participant could answer the included items. A 15-min site observation period was added to site visits to allow the site clinician to observe the participant's interactions and gain information to help answer items on the ABI-S.

### Biosensors

The B-Alert X24 and Q-sensor used in this study were not included in the next study. The B-Alert X24 was generally unable to reach acceptable levels of impedance in children at any site, and there were difficulties with wireless syncing. The impedance issues might relate to difficulties experienced by sites in measuring, creating, and positioning the device with children. These resulted in no complete EEG data sets being obtained. A different wired device, available in multiple cap sizes (making it more adaptable for use in both younger and older participants with varied head size) and with prior thorough testing on children (Luchinger et al., 2011; Prehn-Kristensen et al., 2013; Leventon et al., 2014; Cowell and Decety, 2015; Cremone et al., 2015; Leventon and Bauer, 2017) was selected to use in subsequent studies. Our results underscored the importance of directly testing all devices thoroughly in the target population, and not relying on success in healthy adults. Additionally, the convenience of wireless connectivity was outweighed by the added failure modes. Device-independent issues included participants': (1) inability to sit still without moving; (2) touching and playing with the EEG cap; and (3) muscular activity when asked to keep their eyes closed during one of the resting state conditions. In addition, we found that attention to stimuli (demonstrated by eye-tracking) dropped over lengthy experiments, and we used this data to make adjustments to the length of the battery.

The Q-sensor is no longer commercially available, and so it was replaced with a different device for the next study. Observations over the course of the study advised placement of any EDA device with dry electrodes on the wrist for at least 30 min prior to starting the task battery to allow stabilization of SCL.

The Tobii eye-tracker, AMI Motionlogger, and Actiwave Cardio ECG provided reliable and valid data, and will be used in the next study.

### JAKE task battery

The Cogstate Computerized Test Battery (2016) presented several technical challenges related to integration with the rest of the JAKE system. Among them were issues related to screen resolution (requiring the experimenter to manually change screen resolution and change the display orientation) and missing timestamps for stimuli onset (which prevented synchronization with other biosensors). Additionally, clinicians administering the tests reported that participants were also generally unable to tolerate the length of the Task Battery when including the Cogstate portion (which added an additional 15 min at the end of the battery). These factors led us to remove Cogstate from the JAKE Task Battery for the next study.

Calibration of the eye-tracker was difficult at times, particularly with younger participants. We determined that individuals with ASD are less likely to pay attention to standard calibration targets such as pulsating circles. This was addressed by changing the participants' calibration target from a red dot to an object of interest (e.g., animated cartoons). Additionally, an auditory cue (the sound of a tinkle-bell) was added to draw the participants' focus to the screen. Additional calibration screens were also added throughout the stimuli sets to increase the chance of *post-hoc* calibration correction and monitoring of overall calibration quality following breaks or periods of inattention. To improve our ability to monitor the reliability of eye-tracking results, a method for automatically recording calibration outcomes was introduced.

Crosshairs, presented between different stimuli to direct participants' attention to the screen, were changed to cartoon characters placed at the screen's center on gray background (or a color adjusted to the background color of the stimuli sequence) to make them more attractive to children. In order to keep participants' attention on the screen during the eyes direct/averted ERP experiment, they were additionally asked to count the number of cartoons of a particular type (e.g., “count the buses”) appearing between stimuli of interest. The presentation time of the stimuli was set to more than 1 s after the presentation of the cartoon to ensure that there was no overlap of brain activity.

Finally, based on clinician feedback and data quality (e.g., attention to the screen), which indicated a degradation of participants' compliance with JBW over the course of the Task Battery, it was shortened, and stimuli were divided among the three sets so at least a portion of data for most stimuli would be obtained even if participants did not complete all three sets. Reward schedules for completion of the battery were discussed with sites, and refined for future studies.

### Future investigation and possibilities

The current study demonstrates the feasibility and usability of deploying a relatively inexpensive system of synchronized sensors to clinical sites typical of those employed in pharmaceutical drug studies. Despite the described difficulties with some of the sensors, the experience of the study has demonstrated the overall practicality of the approach and also enabled necessary changes to be made to ensure future success in data collection. Two of the three sites had no experience with EEG, yet, after training, were able to administer the JBW and transmit useable data that could be incorporated into typical data processes that are used in large interventional studies. In general, the lack of reliance on external vendors for collecting and transferring data can dramatically increase the speed of data collection and analysis. The system is thus practical for use in sponsored pharmaceutical studies conducted at distributed clinical sites and does not require a highly specialized network. Experience from the study also demonstrates that children and adults with a range of ASD severities (range of CARS-2 total score: 24–62, VABS total score: 24–111) can tolerate 30–45 min duration of the JBW.

Future refinements of the JAKE system will include use of combinatorial processes and machine learning approaches to search for new features and associations. For example, the degree of search (limited frequency of eye position changes) or pattern (cyclic or repetitive nature) of search in VET may relate to restricted and repetitive behaviors (RRBs) overall. And, the emergence of other types of dynamic task/sensor combinations (e.g., face/construct valid tasks, games with incidental findings, passive recording approaches that allow for summation of large patterns of data) may further enhance the utility of the JAKE system for clinical trials of ASD. Continuing improvements in sensor technology may also allow for more effective measurement of EDA with wrist-based devices and for home-based JBW administration.

The JAKE system continues to be improved upon and is currently being validated in a larger observational study, collecting more robust data with the optimized system. Together with the ABI (Bangerter et al., 2017), we plan to expand use of the JAKE system to interventional studies, with the ultimate goal of making it available so that increasingly more therapeutic candidates can be sensitively, objectively, and rapidly tested to identify efficacious interventions for the growing ASD population.

## Conclusions

The JAKE system aims to identify biomarkers that can stratify individuals with ASD into homogeneous sub-populations and to quantifiably and reproducibly measure intervention outcomes (i.e., improvements of deficits/symptoms in ASD). To this end, advances in biosensor devices and the JAKE portal software and processes, the Task Battery, and the RDW were accomplished over the course of this study. Despite the exploratory nature of this program of research, and current findings limited by small sample sizes and missing data, the JAKE system appears to be a viable platform for use in clinical trials of ASD, with no safety issues observed, and holds promise in identifying potential markers of subpopulations and/or measuring change in ASD.

### Clinical significance

This line of research, utilizing multiple sensors and challenge tasks and from multiple groups [EU-AIMS^1^; ABC-CT^2^], could lead to the identification of biomarkers that can stratify individuals with ASD into homogeneous sub-populations and to quantifiably and reproducibly measure intervention outcomes (i.e., improvements of deficits/symptoms in ASD). This will enable the conduct of high-quality pharmaceutical studies to bring treatments for the core symptoms of ASD to the public.

## Author contributions

SN, GP, AB, NM, AS, MB, GD, YJ, MG, RH, BL, FS, WC, DL, and SJ were involved in study design and/or data collection. DL and SJ were responsible for the statistical analyses. SN, GP, AS, NM, and AB were involved in data analysis. All authors were involved in interpretation of the results and review of the manuscript.

### Conflict of interest statement

Authors SN, NM, AB, DL, SJ, MB, AS, WC, and GP are employees of Janssen Research & Development, LLC. GD is on the Scientific Advisory Boards of Janssen Research and Development and Akili, Inc., a consultant to Roche, has received grant funding from Janssen Research and Development, LLC and PerkinElmer, and receives royalties from Guildford Press and Oxford University Press. MG has received research and consulting funding from Janssen Research & Development, LLC. RH is on the Scientific Advisory Boards of BioMarin Pharmaceutical Inc., Neuren Pharmaceuticals Limited, and Janssen Research and Development, LLC and has research grant funding from Curemark, Roche, Shire, and Sunovion Pharmaceuticals, Inc. BL has received research grant funding from the NIH, is a consultant to Janssen Research and Development, LLC and the Illinois Children's Healthcare Foundation, and is a board member of the Brain Research Foundation. FS has received research funding from Janssen Research and Development, LLC and Roche. The other author declares that the research was conducted in the absence of any commercial or financial relationships that could be construed as a potential conflict of interest.

## Footnotes

^1^European Autism Interventions - A Multicentre Study for Developing New Medications (EU-AIMS). Available online at: http://www.eu-aims.eu/# (Accessed November 7, 2016).

^2^Biomarkers Consortium - The Autism Biomarkers Consortium for Clinical Trials. Available online at: http://www.fnih.org/whatwe-do/current-research-programs/biomarkers-consortium-asd (Accessed November 7, 2016).

## Abbreviations

ABI: Autism behavior inventory
ADOS: Autism diagnostic observation schedule
API: Application protocol interfaces
ASD: Autism spectrum disorder
CARS-2: Child autism rating scale 2
CSV: Comma-separated values
eCRF: electronic case report form
ECG: Electrocardiography
EDA: Electrodermal activity
EEG: Electroencephalography
EMR: Electronic medical records
HAI: High autism interest
HR: Heart rate
HRV: Heart rate variability
IQ: Intelligence quotient
IRB: Independent review board
JAKE: Janssen autism knowledge engine
JBW: JAKE biosensor workbench
K-SADS-PL: Kiddie-SADS-Present and Lifetime version
LAI: Low autism interest
RDW: Research data warehouse
RRB: Restricted and repetitive behaviors
SCL: Skin conductance level
SCQ: Social communication questionnaire
SCR: Skin conductance responses
TD: Typically developing
UI/UX: User interface/User experience
VABS-II: Vineland adaptive behavior scale II
VET: Visual exploration task.
